# Impact of COVID-19 on Emergency Medical Services for Patients with Acute Stroke Presentation in Busan, South Korea

**DOI:** 10.3390/jcm11010094

**Published:** 2021-12-24

**Authors:** Jiyoung Kim, Choongrak Kim, Song Yi Park

**Affiliations:** 1 Department of Neurology, Pusan National University School of Medicine, Busan 50612, Korea; bijoukim78@gmail.com; 2 Sleep Disorder Center, Bio Medical Research Institute, Pusan National University Hospital, Busan 49241, Korea; 3 Department of Statistics, Pusan National University, Busan 46241, Korea; crkim@pusan.ac.kr; 4 Department of Emergency Medicine, Dong-A University Hospital, College of Medicine, Dong-A University, Busan 48114, Korea

**Keywords:** COVID-19, emergency medical services, stroke

## Abstract

The purpose of this retrospective observational study was to identify the impact of COVID-19 on emergency medical services (EMS) processing times and transfers to the emergency department (ED) among patients with acute stroke symptoms before and during the COVID-19 pandemic in Busan, South Korea. The total number of patients using EMS for acute stroke symptoms decreased by 8.2% from 1570 in the pre-COVID-19 period to 1441 during the COVID-19 period. The median (interquartile range) EMS processing time was 29.0 (23–37) min in the pre-COVID-19 period and 33.0 (25–41) minutes in the COVID-19 period (*p* < 0.001). There was a significant decrease in the number of patients transferred to an ED with a comprehensive stroke center (CSC) (6.37%, *p* < 0.001) and an increase in the number of patients transferred to two EDs nearby (2.77%, *p* = 0.018; 3.22%, *p* < 0.001). During the COVID-19 pandemic, EMS processing time increased. The number of patients transferred to ED with CSC was significantly reduced and dispersed. COVID-19 appears to have affected the stroke chain of survival by hindering entry into EDs with stroke centers, the gateway for acute stroke patients.

## 1. Introduction

Early recognition of the symptoms of acute stroke and admitting a patient to a capable stroke center within the golden hour are closely related to a patient’s outcomes and are thus highly emphasized [1]. This series of steps—the recognition of stroke signs and symptoms, the activation of emergency medical services (EMS), prompt transport and prehospital notification to a stroke center, immediate emergency department (ED) triage and evaluation, diagnosis and decision about appropriate therapy, and administration of appropriate drugs or other interventions—is called the stroke chain of survival [2].

A number of studies have examined the factors contributing to the stroke chain of survival, especially delays before and after arrival at the ED in the treatment of stroke patients [3,4,5]. Several studies strongly suggest that the use of EMS is an important modifiable determinant of delay time for acute stroke treatment [6,7]. Furthermore, a study reported modestly lower mortality and more frequent use of thrombolytic therapy in patients with acute ischemic stroke when they were admitted to a designated stroke center [8]. Therefore, the use of EMS by a patient and transport to an ED with a stroke center by EMS providers can be important factors influencing the treatment outcomes of patients with acute stroke symptoms.

However, since the World Health Organization (WHO) declared the coronavirus disease-19 (COVID-19) a pandemic on 11 March 2020, there have been changes in the EMS system for emergency diseases, such as out-of-hospital cardiac arrest and acute myocardial infarction [9,10]. The stroke chain of survival is not excluded from these changes [11,12]. In a study of a stroke network in Europe, it was reported that the mean number of patients with stroke codes dispatched to the hospital by EMS decreased from 78% to 57%, and the time of stroke unit arrival from symptom onset was delayed over 30 min. The authors reported that the access and delivery of patients to secure stroke centers were two major challenges, resulting in a break in the stroke chain of survival [13].

South Korea has had a relatively low incidence of COVID-19 thus far [14]. Nevertheless, several changes have occurred in the EMS system in accordance with strict quarantine guidelines and social distancing strategies [15]. However, there are few studies on which changes have occurred in the EMS system related to the stroke chain of survival amid the COVID-19 pandemic. Therefore, we investigated which changes have occurred in the EMS system for patients with acute stroke presentations amid the COVID-19 pandemic. The purpose of this study was to identify the impact of COVID-19 on EMS processing time and transfers to the ED in patients with acute stroke symptoms.

## 2. Materials and Methods

### 2.1. Study Design

This was a retrospective observational study comparing EMS processing time and identifying the changes in EDs to which adult patients were transported for acute stroke symptoms before and during the COVID-19 pandemic in Busan, South Korea.

The inclusion criteria were all patients transferred to the ED with EMS activation due to acute stroke symptoms during the study period. Acute stroke included both ischemic and hemorrhagic stroke, which we defined as sudden onset of the following symptoms; we set the timeframe of symptom presentation to within 1 week. The included acute stroke symptoms were any lateralizing signs or focal neurologic deficits such as hemiparesis, hemisensory deficit, monoparesis, dysarthria, aphasia, facial palsy, or ataxia. In the case of altered mental status, if there was any additional information about lateralizing signs or focal neurologic deficits, we included it.

The exclusion criteria were patients under the age of 18 and patients with symptom presentation after trauma. We excluded patients with altered mental status alone without any additional information about lateralizing signs or focal neurologic deficits. Thus, we excluded patients with altered mental status due to hypoglycemia, shock, respiratory distress, seizure, fever, or unknown etiology. The reason for exclusion regarding altered mental status with unknown etiology was because acute stroke could not be differentiated from drug intoxication and metabolic encephalopathy in the prehospital stage. We also excluded visual symptoms suggestive of stroke, such as sudden loss of vision, visual field defects, and diplopia, because they were not described in precise terms in the EMS records. We excluded quadriplegia because it was recorded indistinguishably in a patient with coma; however, we included quadriplegia without altered mental status. We did not include vertigo, as most instances were recorded in vague terms such as dizziness.

We defined the period before the COVID-19 pandemic (pre-COVID-19) as 1 March 2019, to 28 February 2020, and the period during the COVID-19 pandemic (COVID-19) as 1 March 2020, to 28 February 2021. Busan reported the first confirmed COVID-19 case on 21 February 2020, and, accordingly, announced a social distancing measure on 24 February 2020, which we referred to as the breakpoint.

### 2.2. Study Setting

Busan, where we conducted this study, is located on the southeastern coast of the Korean peninsula. This region consists of 1 district (gun) and 15 districts (gu), with a population of 3.35 million and an area of 765.94 km^2^ [16].

The EMS system of Busan, the same as that of greater South Korea, is government-based and single-tiered and provides a basic to intermediate level of EMS from fire agency headquarters. As of 2021, the Busan EMS system consists of 1 headquarters, 1 fire school, 11 fire stations with 59 safety centers, and 70 EMS teams. There is one EMS system control center, and all emergency calls are processed and dispatched to the EMS teams. Most EMS teams consist of three EMS providers, usually including at least one emergency medical technician (EMT). Most EMS providers have registered nurse or first/second grade EMT certifications. EMS providers are trained to use the Los Angeles Prehospital Stroke Scale on patients with acute stroke symptoms at the scene, but their skills have not yet been verified. Ambulances with physicians are not available. There are three levels of EDs according to their capabilities: regional emergency centers, local emergency centers, and local emergency departments.

In February 2020, the first confirmed case of COVID-19 in Busan was reported, and changes have since been made in the practice of EMS to reduce the exposure of EMS providers to COVID-19. In the prehospital stage, EMS providers were recommended to use personal protective equipment (PPE) on all dispatches according to the guidelines [17]. In the hospital stage, the ED strengthened the screening of patients with COVID-19-related symptoms and preemptive isolation through triage, and prohibited the allocation of additional beds in the ED, which was previously allowed in overcrowded EDs.

There are five university hospitals (A, B, D, E, and F) in the region and 1 university hospital (G) at the regional border. Of these, hospitals A and G have regional emergency centers, and hospital D operates regional trauma centers. In addition, hospital A has a comprehensive stroke center (CSC). CSCs are equipped with advanced imaging techniques, including MRI/MRA, have experts trained in vascular neurology, endovascular interventions, and neurosurgery for 24 h and are certified by the state [18]. Hospital M is a municipal medical center and was designated a dedicated COVID-19 hospital after the start of the COVID-19 pandemic.

### 2.3. Data Sources and Collection

Prehospital data on all EMS dispatches are electronically collected and managed by regional fire agencies from scene-dispatched EMS providers. For selected cases (out-of-hospital cardiac arrest, suspected cerebrovascular disease, and cardiovascular disease), the EMS providers additionally file and submit a prehospital patient report. Thus, in the dispatch for a patient with acute stroke presentation, EMS providers make two records: one emergency dispatch record (ED record), which contains the detailed EMS processing time, and a cerebrovascular patient report (CV report), which contains the patient’s symptoms and signs. For this study, we collected anonymous prehospital data (ED records and CV reports) from the headquarters of the national fire agency.

For the data collection, we used age and sex as the patient variables and EMS processing time and the number of patients transferred to EDs as the EMS variables. We defined EMS processing time as the time from the patient’s call to ED arrival, consisting of four periods: call time, response time, time at the scene (scene time), and transport time. We defined EMS call time as the time from the start of the call to the end. We defined EMS response time, scene time, and transport time as the time elapsed from the end of the call to EMS arrival at the scene, from EMS arrival at the scene to EMS departure from the scene, and from EMS departure from the scene to EMS arrival at the ED, respectively.

**Figure 1:**
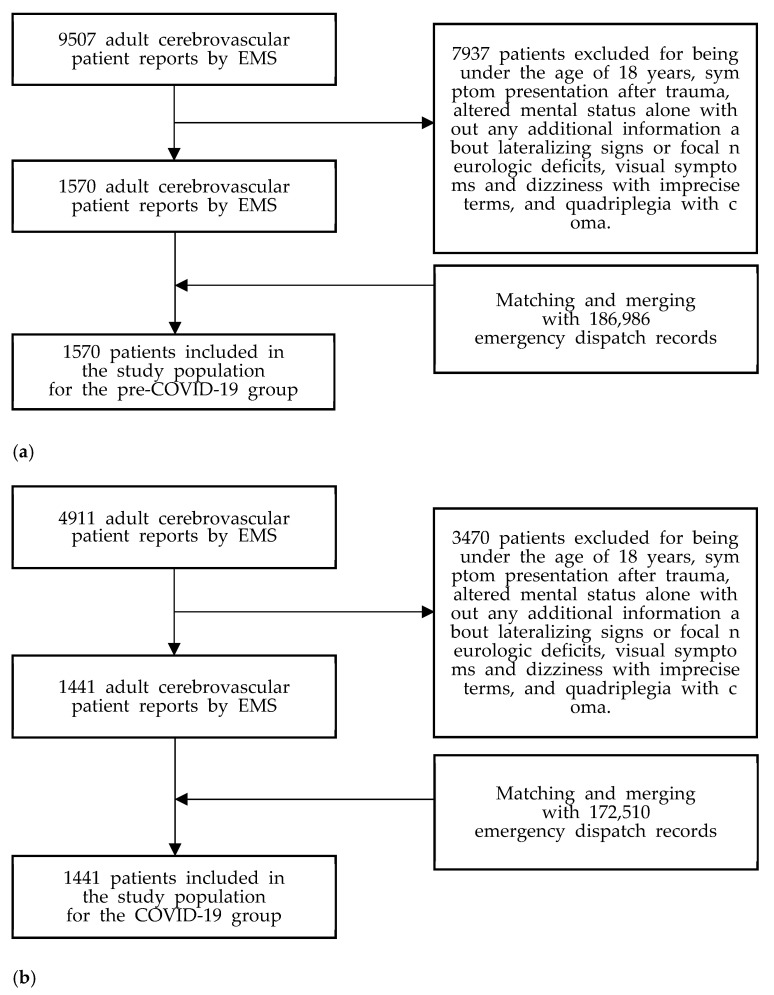
The flowchart of the study population. EMS; emergency medical service, COVID-19; coronavirus disease-19. (**a**) The flow chart for the pre-COVID-19 study population. (**b**) The flow chart for the COVID-19 study population.

We gathered a total of 9507 and 4911 adult CV reports during the pre-COVID-19 and COVID-19 periods, respectively. Of the CV reports, we only re-extracted the reports of patients with an acute stroke presentation via manual search according to the inclusion and exclusion criteria. Since the CV report does not have detailed EMS processing time information, we matched and merged the extracted patient report groups with the ED records one by one. Finally, we included 1570 and 1441 patients in the pre-COVID-19 and COVID-19 study groups, respectively (Figure 1).

### 2.4. Outcome Measures

The primary outcome was the change in EMS processing time, and the secondary outcome was the change in the number of patients transferred to EDs before and during the COVID-19 period.

### 2.5. Statistical Analysis

We performed descriptive analysis to examine the distribution of the variables. Continuous variables are presented as the mean and standard deviation (SD) or median and interquartile range (IQR), while categorical variables are presented as frequencies and proportions. In the comparison of the two groups, we assessed differences in continuous variables using the independent t-test or the Mann–Whitney test; we assessed differences in categorical variables using the chi-square (χ2) test as appropriate. We performed all statistical analyses using SPSS 26.0 (SPSS, Inc., Chicago, IL, USA) and SAS 9.4 (SAS Institute Inc., Cary, NC, USA). We considered a two-sided *p* value of <0.05 to be statistically significant.

## 3. Results

**Figure 2:**
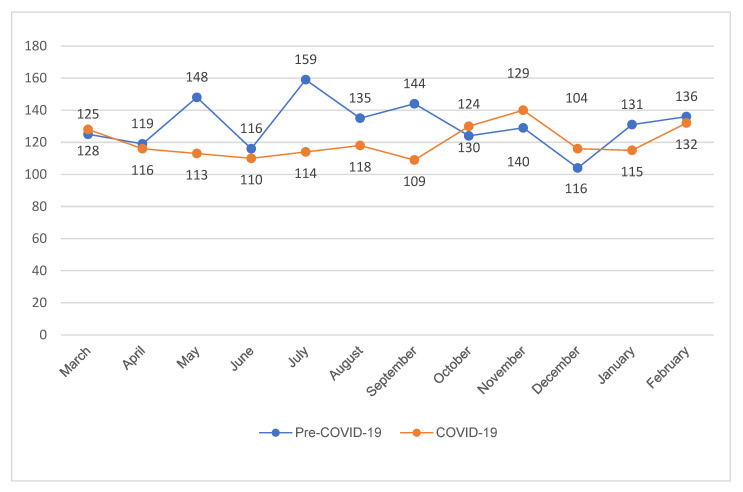
The monthly number of patients using EMS for acute stroke symptoms. COVID-19; coronavirus disease-19.

The total numbers of patients using EMS for acute stroke symptoms were 1570 (pre-COVID-19) and 1441 (COVID-19). The monthly number of patients who required EMS for acute stroke symptoms is shown in Figure 2. The mean age and male sex proportion were 68.0 ± 13.6 years and 52.3% in the pre-COVID group and 69.4 ± 27.7 and 47.7% in the COVID group, but the difference was not statistically significant for age (*p* = 0.072) or sex proportion (*p* = 0.883).

### 3.1. Comparison of EMS Processing Time for Patients with Acute Stroke Symptoms before and during COVID-19

All EMS processing times, except for the call time, were significantly delayed in the COVID-19 period (Table 1).

### 3.2. Comparison of the Number of Patients with Acute Stroke Symptoms Using EMS Who Were Transferred to EDs before and during COVID-19

**Figure 3:**
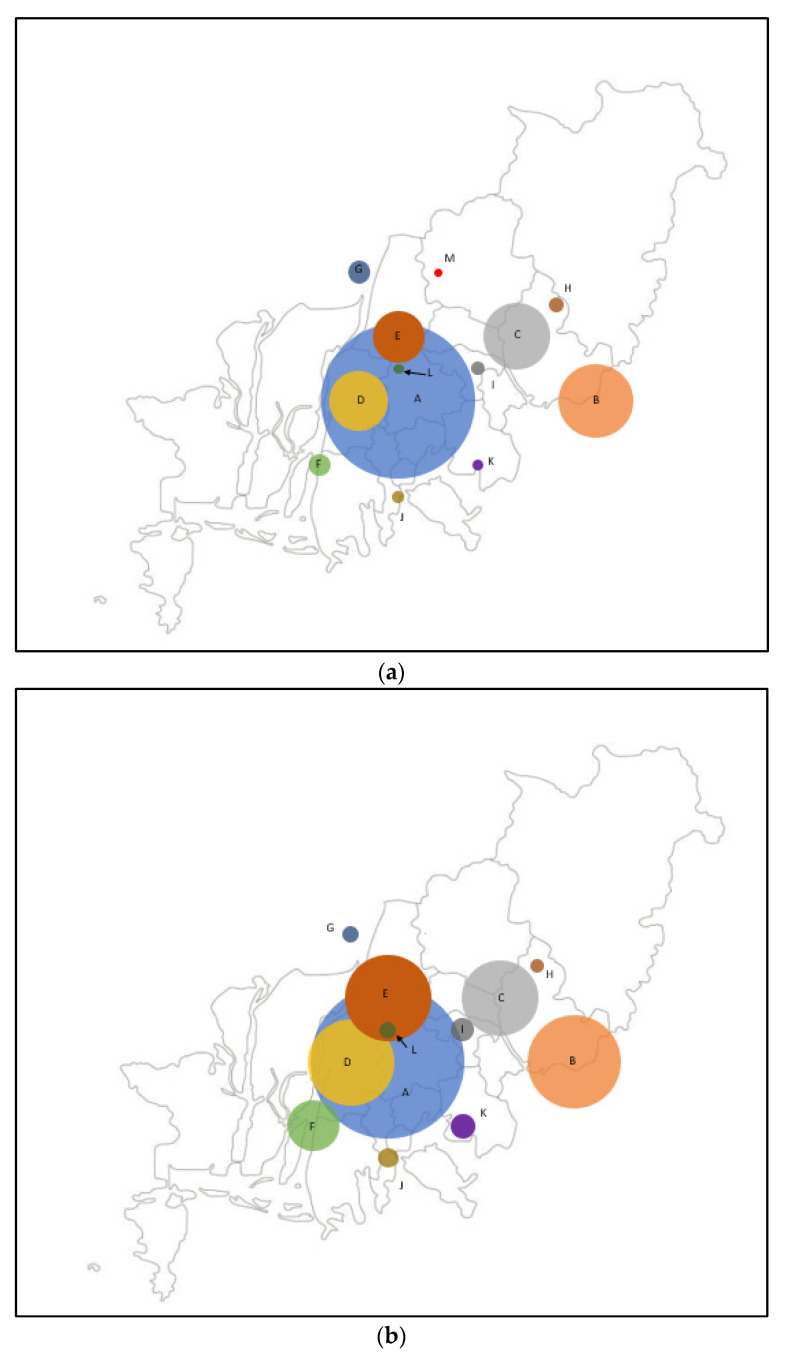
Comparison of the number of patients with acute stroke symptoms using EMS who were transferred to EDs before and amid COVID-19. The diameter of the bubble is proportional to the number of patients transferred to the ED. The color of the bubble for each ED is as follows: A (blue), B (apricot), C (gray), D (mustard), E (brick), F (chartreuse), G (deep blue), H (ochre), I (deep gray), J (camel), K (violet), L (dark green), and M (red). A, C, D, E, F, and G are university hospitals. A has a stroke center. A and G have regional emergency medical centers. M was designated as a hospital dedicated to COVID-19 after the COVID-19 pandemic. ED; emergency department, EMS; emergency medical service, COVID-19; coronavirus disease-19. (**a**) Bubble figure of the number of patients with acute stroke symptoms using EMS who were transferred to EDs before COVID-19. (**b**) Bubble figure of the number of patients with acute stroke symptoms using EMS who were transferred to EDs during COVID-19.

There were significant changes in the number of patients transferred to EDs (Table 2, Figure 3).

## 4. Discussion

This study presents the changes in EMS processing times and transfers to EDs for adult patients with acute stroke symptoms who used EMS before and during the COVID-19 pandemic in Busan, South Korea. The total number of patients using EMS amid COVID-19 declined by 8.2% compared to the pre-COVID-19 period, and the ED processing time was delayed by four minutes. The number of transfers to EDs with a CSC was reduced significantly, and these patients were dispersed to nearby EDs. While many studies have tracked the time delay of inpatient stroke pathways during COVID-19, our study had a strength in focusing on the prehospital stroke pathway in patients with acute stroke symptoms [19].

During the pandemic, the number of patients with acute stroke symptoms transferred to EDs by EMS has varied by country. A study from Germany reported that the referral of stroke codes by EMS has remained stable even during the COVID-19 pandemic lockdown [20]. Canada has seen a 20% drop, and Ohio in the northeastern US has witnessed an approximately 30% decrease [21,22]. These changes appear to be due to differences in the capacity of the EMS system and public awareness and willingness according to the volume of confirmed COVID-19 patients.

We found delays in all EMS processing times except call times before and during the COVID-19 pandemic. An approximately one-minute delay in EMS response time was considered the time it took EMS providers to put on PPE, and an approximately two-minute delay in EMS scene time was considered the time it took to screen patients for COVID-19-related symptoms. EMS response time delay is a change reported in several studies since the COVID-19 pandemic [23]. Hence, except for the essential time related to COVID-19, the EMS processing time in this study is considered not to be significantly delayed compared to that in the pre-COVID-19 period, which means that the EMS system in the region has been operating stably during the COVID-19 pandemic.

Many studies have shown that the hospital arrival time after stroke symptom onset has become delayed during the COVID-19 pandemic. A study from the US reported that the mean time of presentation to the stroke center from the last known well timepoint has become delayed significantly in the COVID-19 period (603 ± 1035 min) compared with the baseline period (442 ± 435, *p* < 0.02) [24]. A study from Hong Kong reported that the median stroke onset to door time has become delayed by over 1 h in the COVID-19 period compared with the pre-COVID-19 period (154 min vs. 95 min, *p* = 0.12) [25]. Delays in time from symptom onset to hospital arrival have also been reported in South Korea (a median time of 91.0 min vs. 176.0 min, *p* = 0.029) [26]. In their study, one cause of the prehospital delay might be that patients may be reluctant to visit the hospital during COVID-19 outbreaks; they did not consider causes for the delay in EMS processing time. Although we did not track the time from symptom onset to hospital arrival, our findings that the EMS system has remained stable even during the COVID-19 pandemic supports their conclusion.

Although many studies have reported prehospital delays, few studies have addressed EMS processing time for patients with acute stroke symptoms. In a study from Spain, there was a substantial increase in the number of EMS calls, and the time from stroke onset to the EMS alert increased by 42 min [27]. However, once EMS were alerted, the EMS system remained stable. Thus, the call time delay was reported as a bottleneck in the regional stroke care system. This call time delay has also been found in France [28]. However, in our study, there was no change in call time. According to our investigation, the number of EMS calls in the region during COVID-19 decreased compared to the pre-COVID-19 period (186,986 vs. 172,510). This reduction may have been a factor in keeping the EMS processing time stable.

We identified three EDs (A, G, M) with significantly reduced EMS transport during COVID-19. Hospital M was designated a dedicated COVID-19 hospital, so no patients other than confirmed COVID-19 patients would have been transferred there. If so, why did the number of patients transported out of EDs A and G decrease? Both EDs are regional emergency centers. In a recent South Korean study by Kim et al. [29], during the COVID-19 period, more acute and severe patients have been concentrated in advanced emergency departments, such as regional emergency centers. The concentration of patients in advanced EDs has resulted in a lack of flow and overcrowding of EDs [30]. We speculate that a similar problem may have occurred in EDs A and G, which are regional emergency centers. In particular, the decline in the number of patients in ED A was the largest. If the CSC of ED A could not accommodate a patient, the ambulance may have been diverted to surrounding EDs E and F. We assume that a similar phenomenon occurred between EDs B and K. ED K is geographically closest to ED B. The number of patients in ED K may have increased due to the blockade of ED B by overcrowding.

Our findings reveal the consequences of centralization. If just one CSC suddenly did not work, a greater risk could arise in patients with acute stroke in the region. As a countermeasure against this, the Italian experience could be helpful. On 20 May 2012, an earthquake struck Modena and Ferrara, causing one of the two hub hospitals to become inoperable. However, the other hospital compensated, which led to maintenance of the medical system [31].

This study has several limitations. First, our study population consisted of patients with acute stroke symptoms; we did not confirm and track whether they were diagnosed with stroke. However, regardless of whether the diagnosis was confirmed, the same criteria should have been applied to patients with acute stroke symptoms in the prehospital stage; thus, this will not be a major limitation in interpreting the results. Second, in selecting the study population, we selected only patients with lateralizing signs or focal neurological deficits. Therefore, we may have excluded those without lateralizing signs or focal neurological deficits who were finally diagnosed with stroke. In addition, there may be some missing patients because we only analyzed the data described by the EMS providers. Third, in patients with acute stroke symptoms, the timing of symptom onset is important, but we did not include it. There were many missing data about onset time in the records of EMS providers, so analysis was not possible. Fourth, we did not follow the prognosis of the patients who were not transferred to CSC. Studies have reported lower mortality in stroke centers [32]; however, this has not yet been validated in this region. Further research on this issue should follow.

In conclusion, during the COVID-19 period, the EMS processing time has increased but has remained relatively stable in the region, except for the time it takes to screen for suspected COVID-19 patients and to put on PPE. However, the number of patients transferred to ED with CSC has become significantly reduced and dispersed. COVID-19 appears to have affected the stroke chain of survival by hindering entry into EDs with stroke centers, the gateway for acute stroke patients.

## Figures and Tables

**Table 1:** Comparison of EMS processing time for patients with acute stroke symptoms before and during COVID-19.

Variables	Study Period	Mean (min)	Q1 (min)	Median (min)	Q3 (min)	*p* Value
EMS processing time	pre-COVID-19	31.3	23.0	29.0	37.0	<0.001
	COVID-19	35.4	25.0	33.0	41.0	
Call time	pre-COVID-19	2.2	2.0	2.0	3.0	0.061
	COVID-19	2.3	2.0	2.0	3.0	
Response time	pre-COVID-19	6.8	4.0	6.0	8.0	<0.001
	COVID-19	7.7	5.0	7.0	9.0	
Scene time	pre-COVID-19	8.0	5.0	7.0	10.0	<0.001
	COVID-19	10.4	6.0	9.0	13.0	
Transport time	pre-COVID-19	14.3	8.0	13.0	19.0	0.034
	COVID-19	15.0	8.0	12.0	19.0	

EMS, emergency medical services; COVID-19, coronavirus disease-19; Q1 and Q3, the first and third quartiles, respectively.

**Table 2:** Comparison of the number of patients with acute stroke symptoms using EMS who were transferred to EDs before and during COVID-19.

ED	Pre-COVID-19		COVID-19		Changes		*p* Value
A	436	(28.15)	311	(21.78)	▼125	(▼6.37)	<0.001
B	211	(13.62)	189	(13.24)	▼22	(▼0.39)	0.799
C	190	(12.27)	154	(10.78)	▼36	(▼1.48)	0.228
D	167	(10.78)	175	(12.25)	▲8	(▲1.47)	0.229
E	148	(9.55)	176	(12.32)	▲28	(▲2.77)	0.018
F	63	(4.07)	104	(7.28)	▲41	(▲3.22)	<0.001
G	63	(4.07)	35	(2.45)	▼28	(▼1.62)	0.018
H	41	(2.65)	28	(1.96)	▼13	(▼0.69)	0.262
I	39	(2.52)	47	(3.29)	▲8	(▲0.77)	0.250
J	34	(2.19)	40	(2.80)	▲6	(▲0.61)	0.345
K	30	(1.94)	50	(3.50)	▲20	(▲1.56)	0.012
L	29	(1.87)	33	(2.31)	▲4	(▲0.44)	0.478
M	23	(1.48)	3	(0.21)	▼20	(▼1.27)	<0.001
Total	1570	(100%)	1441	(100%)			0.077

Variables are presented as numbers (percentages). ED, emergency department; COVID-19, coronavirus disease-19. EDs with less than 1% of patients with acute stroke presentation were omitted. A, C, D, E, F, and G are university hospitals. A has a stroke center. A and G have regional emergency medical centers. M was designated as a hospital dedicated to COVID-19 after the COVID-19 pandemic.

## Data Availability

Raw data were generated at national fire agencies in Korea. Derived data supporting the findings of this study are available from the corresponding author (SYP) upon reasonable request.
